# Mimicking family like attributes to enable a state of personal recovery for persons with mental illness in institutional care settings

**DOI:** 10.1186/s13033-015-0022-x

**Published:** 2015-08-18

**Authors:** Vandana Gopikumar, Kamala Easwaran, Mrinalini Ravi, Nirmal Jude, Joske Bunders

**Affiliations:** The Banyan, 6th Main Road, Mogappair ERI Scheme, Mogappair West, Chennai, 600–037 Tamil Nadu India; Faculty of Earth and Life Sciences (FALW), Athena Institute for Research on Innovation and Communication in Health and Life Sciences, VU University Amsterdam, De Boelelaan 1085, 1081 HV Amsterdam, The Netherlands)

**Keywords:** Homelessness, Mental ill health, Recovery based approaches, Family

## Abstract

**Background:**

The convergence between mental ill health and homelessness is well documented, but critical events that precipitate the downward spiral into homelessness, and promote personal recovery remain only partially explored in India.

**Aims:**

To explore causative factors of the descent into homelessness, and gain insight into creative and innovative approaches that promote personal recovery, specifically in institutional care settings.

**Methods:**

This qualitative study used focus group discussions, detailed personal interviews and anonymised data drawn from patient files. The data were analysed using phenomenological approaches.

**Results:**

Findings suggest that besides poverty and deprivation, death of the primary caregiver is a critical event in precipitating distress and a breakdown in the family, leading to a loss of support systems and a sense of belongingness, and rendering persons with mental illness homeless. Social affiliations, kinship, congruence between the real and ideal self, and the drive to assume a more powerful identity and/or pursue self-actualisation emerged as key factors aiding personal recovery. In the absence of a family, mimicking its attributes appears to ground institutions and professionals in an ethos of responsiveness and user-centricity, thereby promoting personal recovery.

**Conclusions:**

This study highlights the critical need to further explore and understand the nature of distress and descent into homelessness, and gain insight into caregiver strain and strategies that can be developed to reduce the same. It further emphasizes the need to shed light on individual strategies that help pursue wellbeing, and delve deeper into the application of value frameworks in institutions and their role in promoting personal recovery among persons with mental health issues.

## Background

The co-occurrence of mental ill health and homelessness represents a persistent and complex social problem. Persons affected by this phenomenon suffer many losses that result in a compromised quality of life. Homeless persons living with a mental illness experience multiple deprivations and are susceptible to further vulnerabilities such as substance abuse, cognitive deficits, depression, physical ill health and a heightened risk of committing suicide [1–3]. The World Health Organization (WHO) describes this group as highly marginalized [4].

Johnson and Chamberlain [5] posit multivariate pathways into homelessness that include the loss of traditional livelihoods, poverty, unemployment, social exclusion, urbanization, changing social institutions (family dynamics), chronic ill health, poor access to health services and out-of-pocket expenditure. Among many critical aspects, family breakdown has been widely accepted as a key factor leading to an individual’s eventual descent into homelessness [6].

A large-scale study conducted in Spain among persons with mental health concerns, sought to understand precipitating factors that led to homelessness. It found that participants viewed their pathways into homelessness as multi-causal—financial instability, the breakdown of familial and social ties, and mental illness were cited as critical events [7]. The study used a Stressful Life Event (SLE) framework in order to determine the relationship between such events and homelessness and revealed that almost one out of every three persons had experienced the death of a first-grade relation (parent, child or intimate partner), and almost two-thirds had experienced the death of a family member or close friend. In examining the causal relationships of the SLEs to homelessness, economic instability followed by the death of family members and close friends feature as prominent and essential factors. Feelings of abandonment and rejection are also perceived as significant triggers.

If family and emerging positive relationships and environments symbolize well-being, we need to understand what exactly they represent. Waldegrave [8] defines a family as any primary intimate group, which through blood ties or intentional commitment identifies itself as such. Family and its breakdown have been observed to influence many other dimensions of life as well. Building positive behaviour that ensures physical and emotional well-being as a result of mirroring positive values [9] is cited as one such. Similarly, in families that exhibit dysfunctional behaviour such as increased aggression, incessant conflict, rejection and coldness, there is a greater risk of psychological problems including aggression, anti-social personality disorder, anxiety, depression and proneness to suicide [10–12].

The aim of this inquiry is twofold—to explore factors that lead to a downward spiral into homelessness, and gain insight into critical personal and organisational elements that contribute to the process of personal recovery.

## Methods

This study employed a qualitative mixed methods design primarily drawing from phenomenological approaches. Qualitative studies seek to understand the ‘how’ and the ‘what’ of phenomena and delve in-depth into meaning—making [13]. Qualitative data-collection techniques can be designed to be participant-led, thus facilitating the creation of a platform to articulate participant-generated perspectives, meanings and values [14]. Due to the sensitive nature of the central inquiry that attempts to capture subjective experiences of the descent into homelessness, and the positive wellness trajectory thereafter, qualitative methods were considered well-suited.

In-depth, qualitative interviews (n = 4) and focus group discussions (FGDs) (n = 23) were conducted with women who had concurrently experienced a state of homelessness and mental illness, and accessed one or more institutional and quasi-institutional services at The Banyan (a registered not for profit organisation in Chennai, Tamil Nadu). The FGDs encouraged a spirit of openness and offered participants a safe, transparent and open space to share their personal experiences. The interviews and FGDs were conducted by a team of researchers, one with master’s level training in social work (n = 6), one with master’s level training in counselling psychology (n = 4), one with a master’s in mental health services research (n = 5), and one with a master’s in development studies (n = 8). The in-depth interviews were conducted by a social work practitioner with over two decades of experience (n = 4). Additionally information to nuance subjective descriptions were drawn from clients’ files and from annual client led audits of the organisation’s services.

To gain a better understanding of organisational strategies that help alleviate distress and promote personal recovery, the researchers sought to explore the ethos and value-framework that guided practice. A representative sample was drawn from the implementing team of mental health professionals (n = 8) comprising social workers, community workers, health care workers, psychologists and psychiatrists.

### Location of study and population

This study was conducted among homeless women with mental illness. They were drawn from a group of clients who accessed mental health services at The Banyan, a non-governmental organization (NGO) based in Chennai, in the Indian state of Tamil Nadu. The organisation was established in 1993, as a shelter for homeless women with mental health issues. It has since, broadened its scope of work, and built diverse response systems to address the needs of persons with mental health issues (men, women and children) living in a state of poverty and/or homelessness. The range of services include emergency and therapeutic care, rehabilitation and day care, long-term care (graded levels of support including shared housing, and group home options), and skills development and employment facilitation. The organisation aligns itself along the wellness and development paradigm, and offers a robust social care programme to supplement clinical care, and currently services a catchment population of approximately 600,000.

The researchers sought to select a diverse mix of participants based on their current living arrangements, socio-economic status, and occupational preference. For this reason, purposive sampling was employed. The 27 participants comprised women who had returned to their families/homes following admission at The Banyan’s institutional care service (n = 10), women who lived in shared housing facilities and earned incentives within the organisation’s vocational training unit (n = 6), women who lived in shared housing facilities and accessed employment outside of the organisation (n = 7). The in-depth interviews (n = 4) were conducted with women who have passed through The Banyan’s ecosystem of care and have achieved a state of personal recovery.

### Instrument and data collection

The interview guide, and probes for focus group discussions were prepared by the researchers, and no external tools were administered. The schedule was constructed to use predominantly open-ended probes so as to gain insight into subjective realities, maximise participant inputs and contribute to inductive theory building. The probes administered to persons experiencing mental ill-health were designed to gain an understanding across three overarching themes: distress occurring at various periods of time, withdrawal and transition (vulnerability living on the margins, and transitions into safe spaces), and coping and aspiration for the future. The schedule was piloted (n = 7), and modifications regarding order of probes, and suitable venues to conduct FGDs were incorporated. All interviews and FGDs were conducted in the regional language (Tamil), and interactions were translated into Hindi/English for some participants (n = 3). The in-depth interviews (n = 4) were conducted across multiple sessions of approximately 1 h each, over a period of 1 year, and FGDs conducted (n = 23), in one sitting of approximately 1 h each. Any additional information or clarifications post initial analysis (listening to audio recordings, and reading transcripts) were sought through further sessions, both telephonic and in person with some participants (n = 12).

### Method of analysis

All interviews and FGDs were audio recorded and transcribed verbatim in the language of the interaction. Those conducted in regional languages (Tamil), were subsequently translated into English, and back-translated into the regional language. Given the critical importance of phrases and words in capturing the essence and nuances of these subjective realities, the researchers took great care to ensure accuracy and fidelity to participant viewpoints. The analysis was conducted using principles of Thematic Analysis [15]. Multiple researchers employed an inductive approach, and coded the transcripts. Emerging codes were collectively collated, examined by the researchers and categorised into themes based on similarities and differences. The study employed guidelines for good practice proposed by several qualitative research theorists. These included a system of rigorous self monitoring, and comprehensive documentation and constant re-evaluation of all phases of the research process to clearly inform the process of analysis and integration into theory [16, 17]. The study additionally employed a robust member checking process to ensure that the researchers’ interpretation stayed true to participant’s original inputs.

### Ethical considerations

This inquiry was approved by The Banyan’s Institutional Review Board (IRB), and all participants were informed about the study and its aims verbally and in writing. Consent was obtained verbally prior to recording the interviews and FGDs. Participants were informed clearly of their right to refrain from answering specific questions or withdraw from the research process at any given time, without compromising their continued access to care or engagement with The Banyan’s services. All data was anonymised, and is stored on the research server at The Banyan Academy of Leadership in Mental Health (BALM).

## Results and discussion

This section aims to cogently articulate the layered and multi-dimensional experiences of persons who underwent a state of concurrent mental illness and homelessness. It has been organised according to principle themes explored through the interviews and focus group discussions. It includes an interpretative analysis that seeks to effectively capture non-verbal cues, integrate field notes and information from client files, and contribute to a rich and nuanced construction of theory.

First, the role of family breakdown in the *descent into homelessness* is examined. In this context, abuse and domestic violence, death of the primary caregiver, parental intolerance of differences, and the caregiver’s inability to cope with multidimensional losses and distress on account of their wards’ mental ill health and other structural barriers such as poverty and deprivation is considered. Next, the *process of personal recovery* is studied and the importance of family or family like attributes in enabling this transition is analysed. Within this recovery framework, the notion of the family and what it signifies is further understood. The development of such facilitative environments and frameworks in mental health institutions are then studied in the context of those who access short or long term care and support. The impact of *mimicking value systems* or approaches to care, akin a family, within therapeutic ecosystems, that foster a sense of belongingness, is then examined. From this emerges a value framework that the organisation used to catalyse recovery, some grounded in Rogerian ideals of genuineness, unconditional positive regard and empathy and others intuitive and non- structured and yet power restorative, ensuring positive and progressive movement from the present self to the ideal self.

### Descent into homelessness

Among many scenarios, what seemed to result in irreconcilable pain and trauma was the *co*-*occurrence of violence (physical and sexual) and loss of the primary confidante* or emotional anchor, almost simultaneously, at an early stage in one’s life.

My dad burnt my mother with kerosene. He used to come drunk and abuse me. He took me to a lodge and sold me off to men. I saw where he put the money. I took it and ran away from home. After that he threw boiling oil on my legs. With that wound I got on the train. I didn’t have food or anything. I came to the railway station and was sleeping on the road. There were men all around leering at me.—(Ms. M)

This experience perhaps compounded the extent of psychological scarring and underlying susceptibility to mental illness, besides rendering Ms. M homeless. She tried holding on, experienced unrelenting abuse and finally fled when her perpetrator also eliminated her only source of hope and security— mother, in a violent attack that the client frequently replays, signifying presence of trauma. This nature of violence and snowballing of distress is extreme and perhaps not as widespread. However, where the notion of everything that a family and supportive relationships stand for is destroyed, the damage usually is lasting, resulting in a breakdown of trust and security. Her auditory and visual hallucinations seem to be an extension of her subjective experience. Though, however distressing the voices, she prefers to live with them and thus in the comfort of a nurturing ideal.

I wake up in the middle of the night almost habitually to speak to my mother. I can see her or/and hear her voice. I don’t ever want this relationship to end. Though it means I cry almost every night, recalling my past.

Distress as a result *of domestic violence, property, interpersonal conflict and stigma* also features prominently among the reasons for this downward spiral.

My husband used to torture me, he didn’t allow me to sleep and I had no peace at all. I couldn’t talk to my children or family or anyone. I attempted suicide several times because of my husband. One day, not being able to stand it, I just left my house and wandered off.

My husband said that they cheated him and married him off to a crazy woman. He used to beat me and I felt like it would be best for everyone if I died.

We all lived together before. We split after a family property conflict.

Whether *financial or interpersonal conflict, loss of control and self*-*esteem, intolerance of differences on the part of the caregivers* and *subsequent rejection*, or structural barriers such as gender, caste or class based discrimination—or in many cases a *cumulative loss* on many of these counts, combined with *loneliness*—many of these conditions could plot a trajectory of withdrawal from society.

The fascination for the male exists at many levels in the Indian society—such as the mother who yearns for a son, or the wife, who prepares herself for marriage to serve her husband etc. Girls are deprived of education, food and other resources so the male can prepare himself to become the family breadwinner. Even traditional rituals around death favour the male. *A female living with a disability is often treated with scant respect and dignity*. A house or shelter does not necessarily connote a home and family. While this is not the norm, such *spurning and non*-*acceptance* could also result in a person’s wandering away. It is therefore critical that the sociology of mental disorders such as cultural and social histories, structures, norms and influences be explored further.

I was hit and abused and thrown out on the roads every day. They’ll never keep me at home. I didn’t know what to do and the only alternative was to just sleep on the road. I even did all the housework, but no one fed me. If I said anything, they only threw me out of the house. In the rain, I slept outside and they didn’t even feed me.

From the caregiver’s perspective, non-compliance with treatment and the family’s code of conduct, *perpetuation of disharmony, aggression and disruptive, impulsive or ‘socially undesirable’ behaviour* are critical pointers determining a descent into homelessness.

She would never stay at home. I tried many times. She’s kind and loving and when she’s here she helps us out. But then she begins to get restless. And picks up quarrels over issues that will not normally affect her. And takes it to an extreme, even abuses and hits me sometimes. I know that she misses her grandmother and runs first to the graveyard to mourn her loss. After that brief period of grieving and solitude, one can find her amidst a crowd, in street corners, soliciting men and dancing to film music blaring from loudspeakers.

These are the thoughts of a caregiver who tried her best to safeguard her daughter, Ms. S, who was affected with mood disorders, from the perils of a life on the streets, though unsuccessfully. While she would visit home from time to time, her primary identity was that of an eccentric, strong and feisty street dancer. No amount of pressure, conditioning or reasoning, or even firm love brought about the change that would provide her with a sense of stability in life. Brought up mostly by her grandmother, Ms. S’s behaviour changes coincided with her grandmother’s death, a critical event that resulted in emotional distancing from her parents.

Some reasons for leaving the family were more frivolous than others.

I fought with my family members and left home on my own. I just boarded a train and didn’t know where I was going.

However, consistently, the death of a loved one seemed to be a predictor for homelessness.

I was well, but my mother passed away. After her death, people took care of me at home, but there was no one to speak to me with love.

My mother passed away while giving birth to me, my father took care of me, then passed away during my childhood, I don’t have any siblings.

Ms. D immolated herself beside her mother’s grave, unable to live a life without the love, concern and tenderness associated with their relationship.

### The process of personal recovery

While a breakdown in family structure and relationships precipitates a downward drift, the aspiration to live a good life and ability to foster social connections seems to define a path to recovery. This serves as a motivation to stay well and persevere in the face of difficult circumstances. Besides instilling a sense of belonging and integration with mainstream society, this also seems to enhance a person’s sense of identity and meaning. The family as a unit seems to help the individual assume the identity of a care provider, presenting a fresh dimension to their otherwise ‘sick’ and ‘dependent’ identity. The sense of responsibility and the need and opportunity to build a life with significant others, encourages the client to plan for the future and derive meaning from these relationships and processes—all critical pathways to self-realisation.

In keeping with Maslow’s theory of self-actualisation, the process of finding meaning through caring relationships seemed to place power in the hands of the client, and offered pathways to self-actualisation and a purposive life, thus aiding in progressive movement towards the notion of the ideal self. Their need for meaning or a sense of purpose appears to stimulate and define their resilience trajectory.

My biggest strength is that in all these problems, I have never ever wanted to die. I feel like I should live. I know I have a disease and I’m taking medicines—**I should live for everyone**.

I want to see **my children** grow up and get married.

I want to make my children study and bring them up to be very **successful**.

I want to save money with **my husband** and build **a house** and **a secure future**.

Whenever I thought I couldn’t go on, I thought of my son, and knew that suicide wasn’t the answer—**I had to live for him**.

Personal recovery is mostly *value*-*centred*, oriented towards choice, awakens a person’s power and encourages personal responsibility. It is focused on the person, promotes strengths, hopes and dreams and fosters empowerment. Its goals are pro-health, choice, transformation and control [18].

Findings echo those of other recovery theorists, and indicate that the concept is not uni-dimensional but can be conceptualised in multiple ways, across various domains. This is illustrated in the quotes above and also evidenced in the analysis of the data from the FGDs. To some, it signifies the return to ‘normalcy’ while to others it could mean living with a disability and yet enjoying a satisfactory quality of life. For instance, articulation of an interest in marriage, love, productive living, or child rearing, seems to indicate a positive inclination and engagement with life and thus a reversal in the process of withdrawing from society. Adopting this framework of recovery, the need to develop an illness-free or more *powerful* identity for oneself—as a mother, carer, hard worker, spouse, friend, partner or as a successful person, kind person, caring person seems to assume significance.

At an organisational level, in the context of The Banyan, in most circumstances, there was an explicitly stated need to reunite with their birth or marital families, to fulfil perceptions of personal recovery. In instances when that was not attainable, it was observed that the organisation used available resources and opportunities to build an alternative sense of family and home, mimicking attributes of a family, as a strategy.

### Re-integration with the family

I want to live my life back home with my family. I know I can rely on The Banyan, but it is not my home.

Of 1758 persons who have accessed mental health care services at The Banyan’s Transit Care Centre, over the last two decades, 1374 returned to their original home. Poverty, multiple deprivations and lack of appropriate care seem to have rendered these women homeless. For those who experienced long term-needs, based on nature of the enduring problem, abandonment or disinclination to return home, other options had to be developed. On some occasions, choices were made not to return ‘home’ as the original source of stress and withdrawal was attributed to a dysfunctional and unkind family and violent relationships, as described earlier. Continued contact with the organization and the sense of dependability that it fostered also helped. Data from The Banyan’s department of monitoring and evaluation indicate that approximately 12% (in keeping with global trends) of persons who entered The Banyan’s system of care require on-going support towards their long-term care needs.

### Mimicking the family

Social networks are described as a nexus of interpersonal ties consisting of family, friends, or other individuals who provide some type of support that ‘leads one to believe that he or she is cared for, loved, valued, and belongs to a network with mutual obligations’ (Milardo [19]: 13, in [20]: 13). Having considered the importance of the family, and notions of emotional security and commitment that it represents, attempts are now made to use this information to develop emotionally sophisticated care, service packages and environments for persons with mental illness.

This led us to a critical question: what are the values and traits that that are therapeutic and best represent a family? In order to answer this, let us first attempt to understand what a family symbolises.

For many people a family symbolizes blood ties and consistent unconditional support that fosters a sense of belonging. It also connotes a sense of commitment and responsibility that results in continued engagement. Even when spurned, many users longed to return to what they thought and knew to be their only natural ally:

I love The Banyan and all of you, but you cannot be my family. Your kindness made me feel better, but I will be happy only when I’m home, even if my family doesn’t love me or rejects me.

Deeper exploration of clients’ lives revealed the need for a known and familiar environment that was neither sterile nor clinical and easy in its flow of conversations, transactions and emotions. An environment that allowed for differences, that balanced love with encouragement, coercion with firmness, promoted independence and stimulated connectedness, responsibility and trust.

**Fig. 1:**
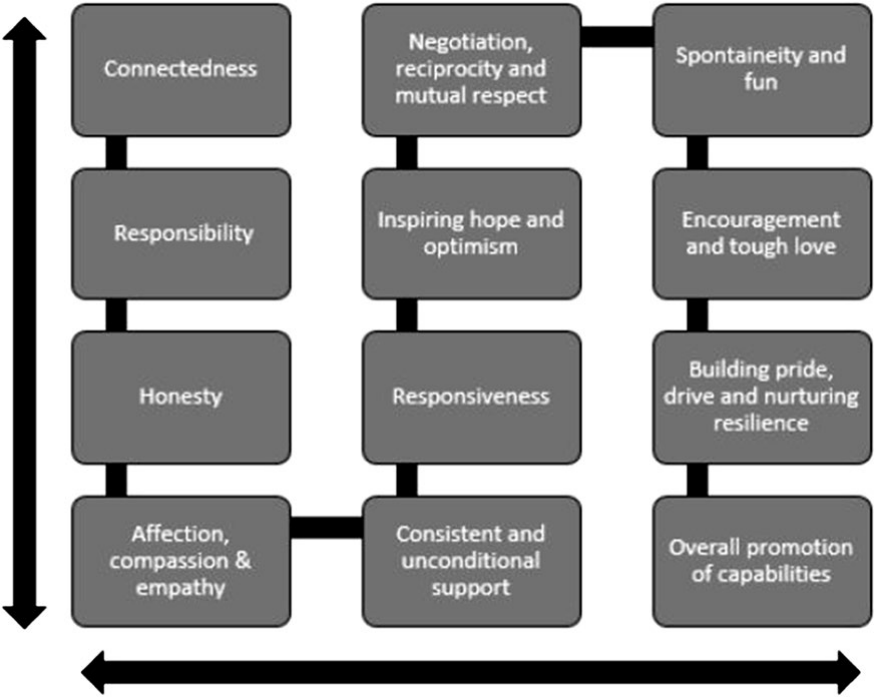
Key values in the process of care as conceptualised by The Banyan.

Based on these expressed needs formulated through an analysis of data emerging from the FGDs, observations and insights of the multi-disciplinary team of mental health professionals at The Banyan, the researchers conceptualized a framework of values that The Banyan employs (highlighted in Fig. 1).

In the context of The Banyan, striking a close and unique bond that moved beyond the technical and therapeutic realm of establishing rapport into something more personal and intimate was observed. Cooking and eating together, decorating the house for functions, welcoming guests, celebrating festivals, mourning the loss of a departed member in culturally relevant and accepted ways, visiting the doctor and hospital together when needed—all denoted commitment and concern, besides connectedness.

The small premises that The Banyan began its operations in resembled a house in a residential area. Owing to a gradually increasing demand, it soon evolved into a larger institution with increased capacity. Coinciding with the development of this new identity, and following the transition from a home to a hospital, there was a need to restructure systems and modify processes. Unlike in the infancy stages, the organization now had to approach issues in a more standardized manner to facilitate scale and growth. Thus, the home-like feel may have been compromised both on account of this transition and an increase in the number of residents. In this learning phase, The Banyan developed the ability to balance the two, realizing the need to continue to mimic the family-based approach and values from the old, intimate house and yet develop institutional and process protocols such that desired impact and reach could be achieved. An effective and appropriate mix of systems and approaches was gradually developed which included fostering therapeutic alliances and ensuring checks and balances in terms of rights, basic care and recovery. In order to translate this framework and ethos into practice, human resource capability within the organisation had to be strengthened.

### Integrative, user-centred approaches

Today, Ms. M (whose quotes was discussed earlier) works for a living and draws an income from various sources—one a government scheme, (the Mahatma Gandhi National Rural Employment Guarantee Scheme), and the other as a house help in the community in which she lives.

I had suffered great loss. Help came from the right source and in a way I could bear to move beyond my painful past and experience. I felt wanted. I felt accepted. I had a group of friends with whom I could celebrate small joys, enjoy an evening of fun at the movies etc. Isn’t that what a family does? I will not go home. I have found work, relationships that matter and a life here. I look forward to my birthday when we cut a cake and I get a gift, (She flashes a broad grin) and every Christmas, when I’m Christmas Thatha (Santa Claus).

Ms. M lives in The Banyan’s Inclusive Ecosystems Programme in a small house rented, by the beach, in Perur, a village in Kancheepuram District in the state of Tamil Nadu. Her day involves all usual chores and activities—cleaning, some cooking, bathing, music in the evenings, some television, a walk to the beach and chats with friends. She now contributes to the economy of the local community through her personal, financial and social transactions and experiences enhanced social mobility. She does, though, continue to experience distress in the nights every now and then but has developed different ways of coping including building resilience systematically and focusing on the positive developments in her life.

Ms. T, on the other hand accessed multiple services at The Banyan, but looking for deeper meaning and contentment hoped to reunite with her son and define her identity as a mother and householder. She had experienced distress on multiple counts, including child sexual abuse, mental ill health, homelessness and the loss of her child while she was living on the street. She showed quick signs of recovery at The Banyan, and was soon working in the housekeeping department for a fixed salary. When The Banyan’s first self-help group was formed, she was elected leader, owing to her entrepreneurial spirit and personal drive. The group soon moved to the clustered group homes in a rural area and subsequently to independent homes in the community. Despite experiencing a good quality of life, she wished to return to familiar surroundings and build a home for herself, in Gudalur, situated in the Nilgiris District, in Tamil Nadu, her place of birth. She continued to maintain contact with The Banyan, both through periods of health and emotional or psychological distress. She was awarded a fellowship^a^ for her work at a mental health outpatient clinic in a non-governmental organisation, located in the vicinity of her home. She has counselled more than 150 people and follows up on their wellbeing telephonically and through home visits, using her story and insights to help others recover. While her mother, brothers and members of The Banyan provide her with support that she values, her primary bond is with her child—this helps her sustain hope, aspire and feel well.

### Implications for research

There is a need for greater understanding related to promotion of recovery, in the Indian context. The role of the family has to then be further explored within this framework. It may also be prudent to test the efficacy of this framework and approach in other contexts. If the results are satisfactory, institutional level reforms could also be guided by similar approaches. Development of protocols may also better enable implementation of a value framework.

### Implication for education

Training of human resources in the health, mental health and social sectors need serious reflection and re-structuring. Current Pedagogy needs to be reviewed and aligned with real world contexts and challenges. The role of reflexive practice and critical thinking in human resource development needs to be reflected adequately in education. While domain based knowledge and multidimensional perspectives and frameworks on mental ill health and recovery are critical, cultivating an environment of continuous learning in the real world becomes equally important.

## Conclusions

With mental ill health and homelessness on the rise, and persistent, unyielding and enduring structural barriers to care exist, it is imperative to understand the aetiology of mental ill health from a sociological and social perspectives as well. This will result in an in-depth understanding of individual perceptions, experiences and notions of distress and wellbeing/state of personal recovery. The critical themes of cumulative loss of agency, control, and belongingness as a key tipping point, and the importance of fostering a sense of meaning, purpose and hope, and nurturing capabilities as key strategies to build resilience emerging from this study, need more focused attention. Understanding value based frameworks of care, and grounding institutions and thus mental health professionals in the same, could have implications that can transform institutional mental health care from a system ridden with stigma, violative and restrictive practices, to one that is responsive, progressive and promotes individual capabilities and personal recovery.

## Endnotes

^a^The BALM fellowship is a leadership and mentoring programme with a stipend awarded by The Banyan Academy of Leadership in Mental Health (BALM) to persons affected by mental health issues, who desire to work within the mental health and development space.

## Abbreviations

WHO: World Health Organisation
SLE: Stressful Life Event
NGO: Non-Governmental Organisation
FGD: Focus Group Discussion
TCC: Transit Care Centre
MGNREGS: Mahatma Gandhi National Rural Employment Guarantee Scheme
